# Estrogen ameliorates allergic airway inflammation by regulating activation of NLRP3 in mice

**DOI:** 10.1042/BSR20181117

**Published:** 2019-01-08

**Authors:** Cheng Cheng, Huimei Wu, Muzi Wang, Lixia Wang, Hongyun Zou, Shuai Li, Rongyu Liu

**Affiliations:** 1 Department of Pulmonary, Anhui Geriatric Institute, The First Affiliated Hospital of Anhui Medical University, Jixi Road 218, Hefei, Anhui 230022, P.R. China; 2 Department of Cardiovascular, The Second Affiliated Hospital of Anhui Medical University, Furong Road 678, Hefei, Anhui 230601, P.R. China

**Keywords:** allergic airway inflammation, asthma, estrogen, estrogen receptor, NLRP3

## Abstract

**Background:** Estrogen has been suggested to play a protective role against airway inflammations, such as asthma. In these processes, the inflammasome nucleotide-binding oligomerization domain, leucine-rich repeat and pyrin domain containing 3 (NLRP3) partly accounts for the activation of pro-inflammatory factors. The aim of the present study was to investigate whether NLRP3 was involved in the protective effect of estrogen against allergic airway inflammation. **Methods:** An ovariectomy was performed on female C57BL/6 mice; some were sham-operated (sham). We then sensitized and challenged them with ovalbumin (OVA) to establish an airway inflammation model. Meanwhile, some mice were treated with 17β-estradiol (E2) for 28 days. **Results:** The expression of NLRP3 inflammasome and its downstream products, caspase-1 and the pro-inflammatory cytokine interleukin (IL)-1β (IL-1β), increased concomitantly with OVA-challenged airway inflammation and decreased with the expression of estrogen receptor β (ERβ). In addition, treating ovariectomized (OVX) mice with E2 dramatically ameliorated airway inflammation via such mechanisms as leukocyte recruitment, mucus production, and secretion of pro-inflammatory cytokines other than IL-18 in bronchoalveolar lavage (BAL) fluid (BALF). Furthermore, E2 suppressed both the mRNA expression and protein expression of NLRP3, caspase-1, and IL-1β. In summary, our study showed that NLRP3 inflammasome activation and pro-inflammatory cytokine production markedly increased in OVA-induced airway inflammation, and E2 effectively abrogated such inflammation by regulating the activation of NLRP3.

## Introduction

Asthma is a chronic disease with high prevalence worldwide. It is characterized by airway inflammation as well as reversible airway obstruction and hyperresponsiveness (AHR) that cause dyspnea, cough, and wheezing [1]. There is a gender disparity: before puberty, girls have lower prevalence and severity of asthma than boys [2], but this trend reverses after puberty. Approximately 30–40% of women are likely to suffer from more severe symptoms and hospitalizations during the pre- or peri-menstrual period because of changes in estrogen and progesterone levels [3,4]. Several studies have demonstrated that between menarche and menopause, females have increasingly severe asthma compared with males [5,6]. After menopause, there is no obvious difference in asthma prevalence between men and women [7]. Furthermore, postmenopausal women treated with hormone replacement therapy obtain ameliorative effects against asthma [8]. Moreover, several animal model studies have suggested that estrogen plays a protective role against allergic inflammation [9–11]. The correlation between sex hormone-modulated inflammation and asthma pathogenesis has become well known.

Estrogen modulates the immune function via its receptors, estrogen nuclear receptors α and β (ERα, ERβ) and G protein-coupled receptor 30 (GPR30), which are expressed by a large number of immune regulatory cells [12,13]. There is evidence that estrogen can induce smooth muscle relaxation in the airway [14], and it may also regulate secretory leukoprotease inhibitor (SLPI) to protect the airway from inflammation in asthma [15]. In addition, estrogen acts on structural cells indirectly affecting lung tissue inflammatory response [16–18]. However, the mechanisms for the association remain largely unclear.

Over the past few decades, growing evidence has indicated that the inflammasome nucleotide-binding oligomerization domain, leucine-rich repeat and pyrin protein containing 3 (NLRP3) plays a critical role in airway inflammations, such as asthma [19,20]. Danger-associated molecular patterns (DAMPs) or pathogen-associated molecular patterns (PAMPs) are signals that promote assembly of apoptosis-associated speck-like protein containing caspase activation and recruitment domain (ARC) and of pro-caspase-1 and then trigger NLRP3. Meanwhile, the Toll-like receptor (TLR)–nuclear factor κ-light-chain-enhancer of activated B cells (NF-κB) pathway up-regulates the expression of pro-inflammatory cytokines interleukin (IL)-1β and -18 (pro-IL-1β and pro-IL-18) [21,22]. Activated NLRP3 cleaves pro-caspase-1 into activated form, which in turn processes the inactivated forms of IL-1β and IL-18 into their activated secreted forms. Recent reports have indicated that NLRP3 level increases during challenge with allergens [23,24], and it has been suggested that NLRP3-specific inhibitors reverse neutrophilic inflammation in a model of allergic airway disease [25]. Emerging evidence has shown that estrogen as an inflammatory protective factor can suppress NLRP3-mediated inflammation in the brain [26,27]. However, the relationship between NLRP3 and estrogen in allergic airway inflammation has not been elucidated.

Given that estrogen can regulate NLRP3 to inhibit pro-inflammatory effects in the brain and other tissues, we hypothesized that it may suppress allergic airway inflammation by regulating NLRP3 activation. To test this hypothesis, we established a model of allergic airway inflammation and ovariectomy (OVX) in mice and then examined activation of NLRP3 and downstream cytokines.

## Materials and methods

### Animals

**Figure 1:**
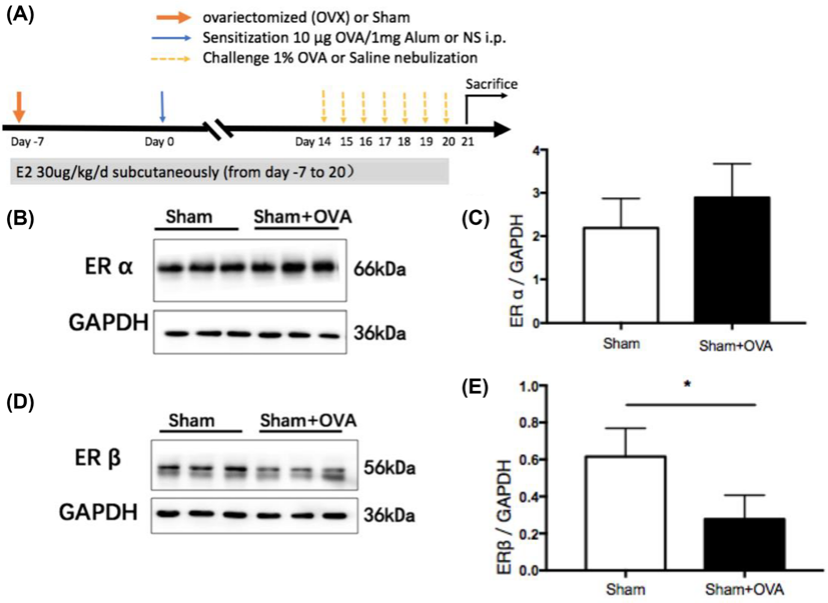
ERβ expression was significantly decreased in OVA mice (**A**) Experimental protocol of the study. (**B**) ERα protein expression in lung tissues of OVA-challenged mice and Sham control mice was evaluated by Western blot. (**C**) The relative level of ERα in lung tissues. (**D**) ERβ protein expression in lung of OVA-challenged mice and Sham control mice was evaluated by Western blot. (**E**) The relative level of ERβ in lung. Data were presented as means ± S.E.M. (*n*=6 in each group). *P<0.05. Abbreviations: i.p., intraperitoneal; Sham, Sham-operated mice.

Thirty 5- to 6-week-old female C57BL/6 mice were acquired that were free of specific pathogens from Shanghai Laboratory Animal Center, Shanghai, China. The mice were housed in a temperature- and light-controlled room and fed a standard diet. All animal experiments were approved by the Committee on the Ethics of Animal Care and Use, Anhui Medical University, Hefei, China. Thirty mice were randomly divided into five groups (each *n*=6): (i) sham operation challenged with saline (sham), (ii) sham operation challenged with ovalbumin (sham–OVA), (iii) ovariectomy challenged with saline (OVX), (iv) ovariectomy challenged with ovalbumin (OVX–OVA), and (v) ovariectomy challenged with both ovalbumin and 17β-estradiol (OVX–OVA–E2). An overview of the protocol is shown in Figure 1A.

### Experimental protocols and treatment

#### Ovariectomy

Female mice were anesthetized by intraperitoneal (i.p.) injection of 1% sodium pentobarbital 8 ml/kg and subsequently subjected to surgical ovariectomy or sham operation via bilateral back incision (day –7). After surgery, thermostatic blanket was used to maintain body temperature until the mice regained consciousness. Sham-operated control mice were subjected to all the same procedures except for ovary removal (sham).

#### OVA exposure

One week after surgery (day 0), mice were sensitized by i.p. injection of 10 μg OVA (Sigma, St. Louis, MO, U.S.A.) complexed with 1 mg potassium aluminum sulphate (Sangon Biotech, Shanghai, China) in 0.5 Ml of saline. Subsequently, mice were exposed to aerosolized 1% OVA for 30 min per day from day 14 to 20 using an ultrasonic nebulizer device [28]. Sham and OVX groups were saline-sensitized and challenged instead of OVA-aluminum solution.

#### E2 treatment

From day –7 to 20, OVX-OVA-E2 group animals were subcutaneously given E2 (Sigma–Aldrich, St. Louis, MO, U.S.A.) at 30 μg/kg/day for 4 weeks. The pulverized E2 was dissolved in absolute alcohol to the concentration of 50 μg/ml, and then diluted in sesame oil (Acros) to the concentration of 5 μg/ml. The OVX-OVA group was subcutaneously injected with the same volume of sesame oil as placebo simultaneously [26].

Twenty-four hours after the last challenge on day 21, the mice were killed. Then serum samples were collected, and the bronchoalveolar lavage (BAL) fluid (BALF) was collected from the left lung. The right lung was appropriately disposed for histopathological, immunofluorescence staining analysis, real-time RT-PCR (Q-PCR), and Western blot assays.

### Determination of cell classification counts in BALF

Immediately after killing, the left lung was lavaged by instilling and withdrawing 0.5 ml of PBS three times via the tracheal cannula. After the collected BALF was centrifuged at low speed (800×***g***, 4°C, 5 min), the cell dregs were resuspended with 200 μl PBS. The total number of cells was determined in a hemocytometer. Differential cell counting was performed by staining cytosine of BALF resuspension using Wright Stain solution (Sigma) to determine the percentages of lymphocytes, monocytes, neutrophils, and eosinophils by counting at least 200 cells. The cell-free supernatant fluids were stored in −80°C and further used for the detection of related cytokines.

### Histology

After the collection of BALF, a part of each right lung was fixed in 4% paraformaldehyde and then processed into paraffin-embedded blocks. The blocks were incised into 5 μm and stained with H&E. To evaluate the pre-bronchial inflammation, the number of rings of inflammatory cells around bronchia was calculated [29]. Mucus-secreting goblet cells were observed by staining with Periodic acid–Schiff (PAS) by dividing the number of PAS+ cells in the airway by the perimeter of the basement membrane (Pbm) [30] ELISA analysis for serum E2 and inflammatory cytokines.

E2 and OVA-specific IgE levels in serum, IL-6, IL-1 β, IL-18, and tumor necrosis factor-α (TNF-α) levels in BALF were measured with an ELISA kit (Cusabio, Wuhan, China) in accordance with the manufacturer’s instructions. Enzymatic activity was determined spectrophotometrically in absorbance at 450 nm using a 96-well plate reader. The detection limit for E2, IL-6, IL-1 β, IL-18, and TNF-α was 25, 1.56, 31.25, 1.56, and 15.6 pg/ml, respectively.

### Total RNA extraction and quantitative real-time RT-PCR

Total RNA from lung tissues was extracted using TRIzol regent (Invitrogen), and then reverse transcribed to cDNA via reverse transcriptase (Promega). PCR primer sequences used in the present study are listed in Table 1 (Sangon Biotech).

**Table 1:** PCR primers

Gene	Forward primer	Reverse primer
*NLRP3*	AGATTACCCGCCCGAGAAAG	TCCCAGCAAACCCATCCACT
*ASC*	GGAGTCGTATGGCTTGGAGC	ACAAAGTGTCCTGTTCTGGCT
*IL-1β*	GCCACCTTTTGACAGTGATG	AAGGTCCACGGGAAAGACAC
*IL-18*	AAGAGGACTGGCTGTGAC	CTCGGGTATTCTGTTATGGA
*β-actin*	CTGTATGCCTCTGGTCGTAC	TGATGTCACGCACGATTTCC

Real-time PCR was performed using SYBR Green Mix (Takara), and analyzed using an RT-PCR instrument (Applied Biosystems, Foster City, CA, U.S.A.). Amplification of β-actin was also measured as an internal control. The quantitation of target gene expression was analyzed by 2^−ΔΔ*C*^_t_ method [31].

### Western blotting analysis

Lung tissues were homogenized in RIPA buffer consisting of protease inhibitor cocktail (Roche) and phosphatase inhibitor PhosSTOP. The homogenate was centrifuged at 12000 rpm for 15 min at 4°C, and the supernatant was extracted for further protein quantitation. Twenty-five micrograms of protein for each sample was separated on 12% SDS/PAGE gel electrophoresis, and transferred to PVDF membrane. The selected membrane was blocked with 5% nonfat milk for 1 h at room temperature with 120 bpm shaking. Subsequently, the membrane was incubated with primary antibodies at a dilution 1:500 to 1:1000 overnight at 4°C. The primary antibodies used are listed: NLRP3 (CST#D4D86, Cell Signaling), ASC (CST# D2W8U, Cell Signaling), caspase-1 (sc-56036, Santa Cruz Biotechnology), ERα (sc-71084, Santa Cruz Biotechnology), ERβ (sc-53934, Santa Cruz Biotechnology), GAPDH (KC-5G5, KangChen) was performed as an internal control. After washing with 1× TBST, the membrane was incubated with secondary anti-rabbit/mouse IgG for 1 h at room temperature. The protein quantitative analysis was done using ImageJ software.

### Immunofluorescence staining and confocal microscopy assay

Paraffin tissue sections were immersed in the EDTA antigen retrieval buffer (pH 8.0) for antigen retrieval in a microwave oven after deparaffinage and rehydration. The sections were blocked with BSA (Servicebio, China) for 30 min, followed by incubation with primary antibody (ASC, Caspase-1, NLRP3) overnight at 4°C. After primary incubation, sections were washed for 3 × 5 min with PBS, and then incubated with the secondary antibody at room temperature for 50 min in dark condition. The sections were then incubated with DAPI solution at room temperature for 10 min to accomplish DAPI counterstain. Confocal laser microscopy (LSM 510, Carl Zeiss, Göttingen, Germany) was used to detect and collect images.

### Statistical analysis

Statistical analysis was performed using version 24 (SPSS Inc., Chicago, Illinois, U.S.A.). Data were presented as means ± S.E.M. The independent two-sample *t* test was performed to investigate the comparison of ERα and ERβ expressions. The one-way ANOVA followed by LSD post hoc test was used to analyze the differences amongst groups. *P*-value <0.05 was considered significant.

## Results

### ERβ expression significantly decreased in OVA mice

To investigate the involvement of estrogen receptors in the lungs of allergic mice, we measured ERα and ERβ protein expression. ERβ protein expression in the lung was significantly lower in OVA mice than in sham mice (Figure 1D,E), but there was no difference in ERα protein expression between the two groups (Figure 1B,C).

### Estrogen replacement increased plasma E2 levels after ovariectomy

**Figure 2:**
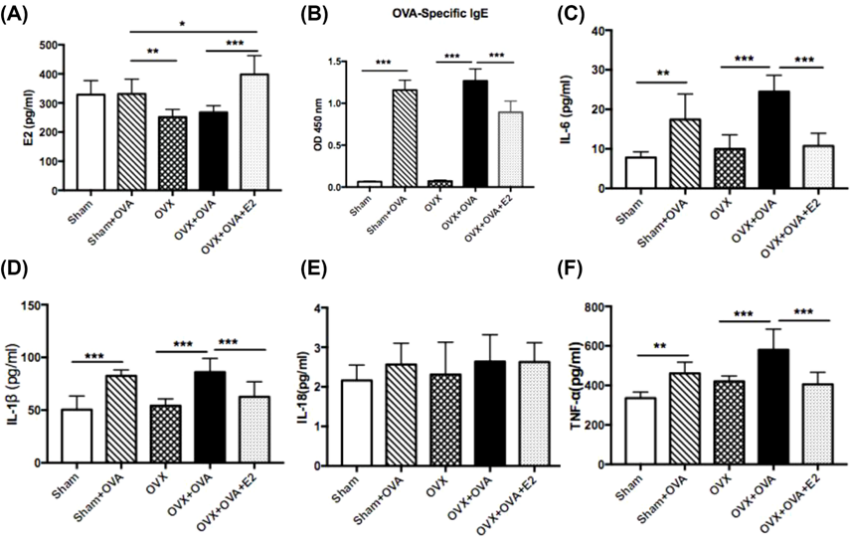
Estrogen suppresses the OVA-challenged IgE in serum and pro-inflammatory cytokines in BALF (**A**) Serum levels of E2. (**B**) Serum levels of OVA-specific IgE. (**C**–**F**) Production of IL-6, IL-1β, IL-18, and TNF-α in BALF. Data were presented as means ± S.E.M. (*n*=6 in each group). *P<0.05, **P<0.001, ***P<0.001.

To verify the success of ovariectomy and estrogen replacement, the level of E2 was measured. Plasma E2 level was not significantly decreased to almost 0 pg/ml after ovariectomy, but it increased dramatically after subsequent subcutaneous estrogen replacement therapy (Figure 2A).

### Estrogen suppressed allergen-induced airway inflammation

**Figure 3:**
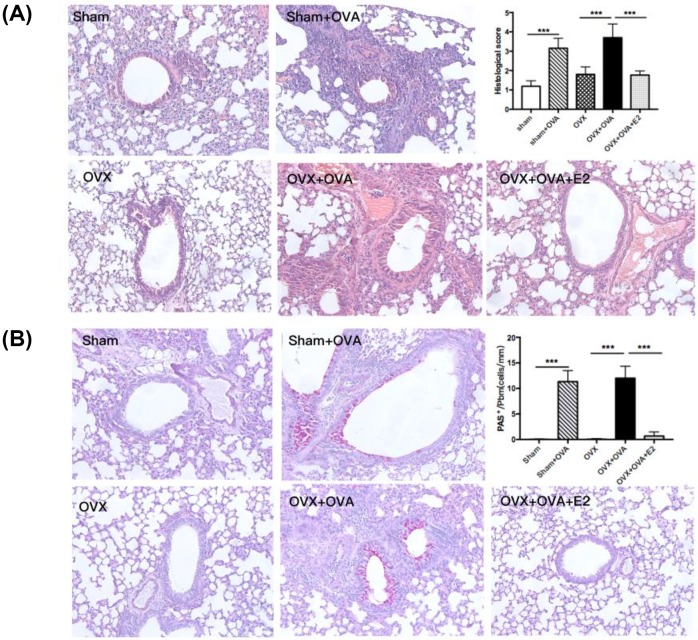
Estrogen significantly inhibits airway inflammation and goblet cell hyperplasia (**A**) Representative H&E-stained sections of lungs (magnification ×20) and histological scoring of lung inflammatory from WT mice. (**B**) Representative PAS staining of lung sections (magnification ×20) and quantitation of PAS+ cells in the airway from WT mice. Data were presented as means ± S.E.M. (*n*=6 in each group). ***P<0.001.

**Figure 4:**
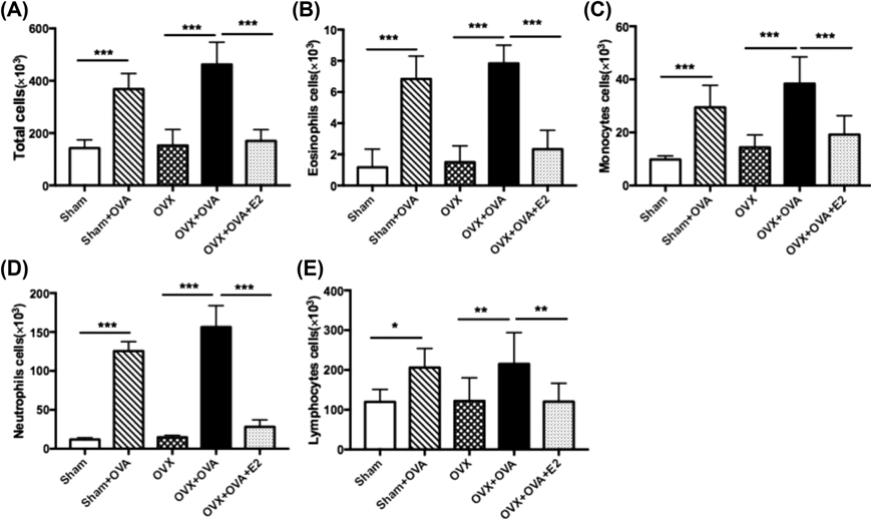
Estrogen reduces the OVA-reduced inflammatory cell numbers in BALF Data shown represent changes in the total number of cells (**A**), eosinophils (**B**), monocytes (**C**), neutrophils (**D**), and lymphocytes (**E**) in the BALF. Data were presented as means ± S.E.M. (*n*=6 in each group). *P<0.05, **P<0.001, ***P<0.001.

Enhanced airway inflammation was evidenced by histopathological characteristics including thickened bronchial wall, peribronchial edema, greater infiltration of inflammatory cells, enfolded epithelium and PAS+ cells surrounding the airway in OVA-sensitized mice compared with the sham and OVX groups. Compared with OVX–OVA mice, the intensity of inflammatory infiltrates and mucus overproduction were dramatically inhibited in OVX–OVA–E2 mice (Figure 3A,B). Consistent with the histopathology, we observed a significant rise in cell numbers, including total cells, eosinophils, monocytes, and lymphocytes, in BALF from both OVA and OVX–OVA mice. However, the increase in the numbers of leukocytes in OVA-induced mice decreased largely in E2-treated mice (OVX–OVA–E2; Figure 4A–E).

### Estrogen reduced levels of serum OVA-specific IgE in allergen-induced mice

Levels of OVA-specific IgE rose dramatically in OVA-induced mice. Moreover, E2-treated mice showed a significant decrease in OVA-specific IgE levels compared with OVX–OVA mice (Figure 2B).

### Estrogen inhibited IL-6, TNF-α, and IL-1β levels in BALF

ELISA analysis showed that levels of IL-6 and TNF-α were significantly increased in OVA-induced mice. After administration of E2, these increases were notably attenuated in OVX–OVA–E2 mice (Figure 2C,F). Importantly, the level of IL-1β cleaved by activated caspase-1 was also considerably reduced in E2-treated mice (Figure 2D). However, there was no difference in levels of IL-18, the other inflammatory cytokine related to NLRP3, amongst these groups (Figure 2E).

### Estrogen suppressed mRNA expression of IL-1β and NLRP3 in lung tissues

**Figure 5:**
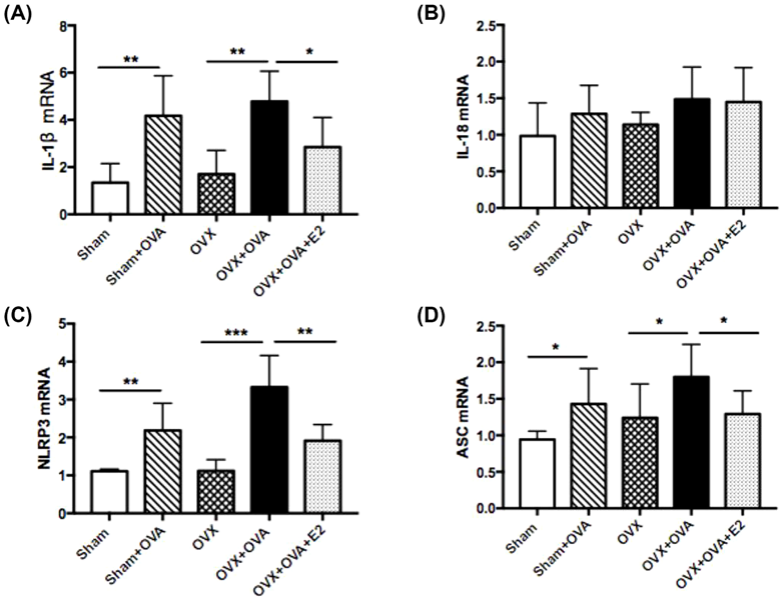
Estrogen suppresses mRNA expression of IL-1β and NLRP3 inflammasome in lung tissues (**A**) *IL-1β* mRNA level; (**B**) *IL-18* mRNA level; (**C**) *NLRP3* mRNA level; (**D**) *ASC* mRNA level. Data were presented as means ± S.E.M. (*n*=6 in each group). *P<0.05, **P<0.001, ***P<0.001.

OVA and OVX–OVA mice showed higher levels of *IL-1β* mRNA expression than did sham and OVX mice. Estrogen replacement reversed the OVA-induced increase in *IL-1β* mRNA expression (Figure 5A). However, there was no significant difference in levels of *IL-18* mRNA expression amongst these groups. E2 treatment did not affect IL-18 levels in OVX–OVA mice (Figure 5B). *NLRP3* and *ASC* mRNA levels were detected in lung tissues using RT-qPCR analysis. As shown in Figure 5C, *NLRP3* mRNA expression was significantly increased in OVA and OVX–OVA mice compared with sham and OVX mice. E2 treatment strongly suppressed *NLRP3* mRNA expression. Likewise, *ASC* mRNA expression levels were significantly increased in OVA-induced mice, and E2 treatment robustly suppressed elevation of *ASC* mRNA (Figure 5D).

### Estrogen decreased protein expression of allergen-induced NLRP3 in lung tissues

**Figure 6:**
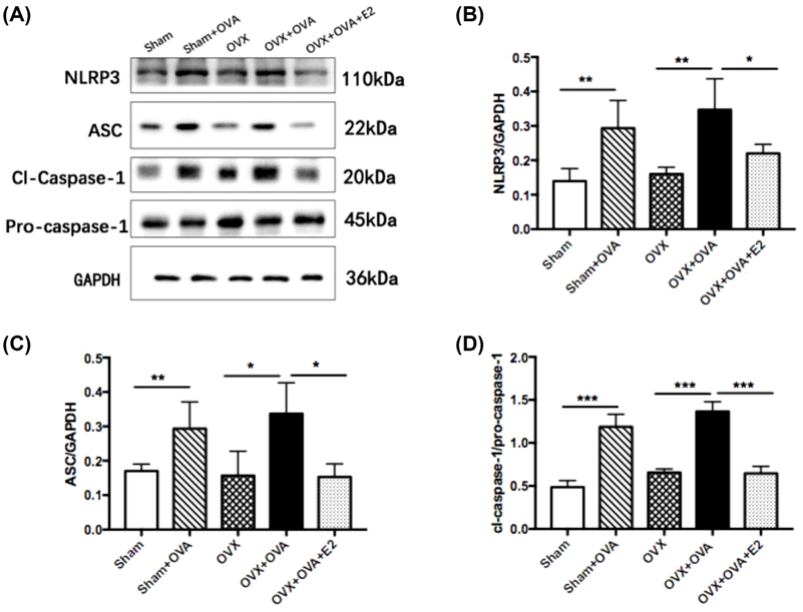
Estrogen decreases protein expression of allergic-induced NLRP3 inflammasome in lung tissues by Western blot (**A**) Representative protein bands of NLRP3, ASC, cl-caspase-1, pro-caspase-1, and GAPDH in the lung tissues. (**B**–**E**) The relative levels of NLRP3, ASC, and caspase-1 in the lung tissues. Gels were representative of at least three independent experiments. Data were presented as means ± S.E.M. (*n*=6 in each group). *P<0.05, **P<0.001, ***P<0.001.

Besides NLRP3 and ASC, caspase-1 is another NLRP3 pathway molecule, and pro-caspase-1 cleaves to active caspase-1. To investigate the protein level of NLRP3 pathway molecules in lung tissues, we performed Western blot analysis. As shown in Figure 6A–D, the results demonstrated that protein levels of NLRP3, ASC, and cleaved caspase-1 (cl-caspase-1)/pro-caspase-1 significantly increased in the OVA and OVX–OVA groups compared with the sham and OVX groups. Furthermore, E2 treatment markedly attenuated the enhanced protein expression of NLRP3, ASC, and caspase-1.

**Figure 7:**
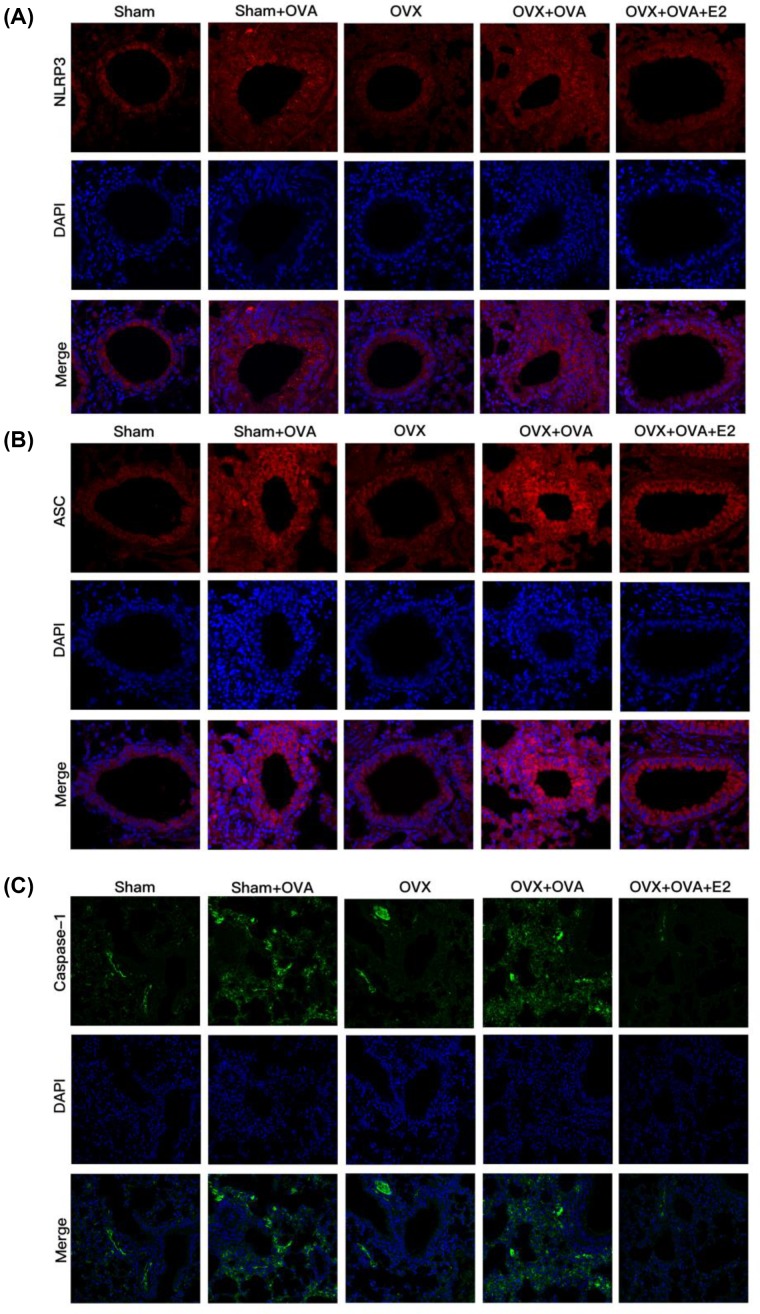
Estrogen attenuates protein expression of OVA-induced NLRP3 inflammasome in lung tissues by immunofluorescence staining (**A**–**C**) The expression of NLRP3, ASC, and caspase-1 in the lung tissues by immunofluorescence via confocal microscope (magnification ×40). Nuclei were stained by DAPI. Gels were representative of at least three independent experiments.

To verify mRNA as well as Western blot results, we further performed IF staining to explore the protein expression of NLRP3, ASC, and caspase-1. All three were highly expressed in OVA-induced mice. Moreover, E2 treatment led to a reduction in such expression in OVA-challenged mice compared with OVX–OVA mice (Figure 7A–C). Combined with our analyses of mRNA expression (Figure 5) and Western blot (Figure 6), these results demonstrated that expression of NLRP3 pathway molecules was increased in OVA-challenged mice and suppressed by E2 treatment.

## Discussion

In the current study, we demonstrated that OVA significantly increased the expression of NLRP3 components, including NLRP3, ASC, and caspase-1. E2 appeared to suppress allergen-induced airway inflammation by regulating NLRP3 activation and pro-inflammatory cytokine production.

Over the last few decades, several studies using animal models have shown that estrogen reduces the associated AHR and inflammation, but the effect is complex, and the mechanism for the association remains obscure. Some studies [9,10] have suggested that estrogen treatment was effective in improving murine allergic asthma and that estrogen seemed to regulate neurokinin-1-dependent prejunctional activation of airway smooth muscles (ASMs) [11]. Similarly, Degano et al. [32] demonstrated that estradiol decreases airway responsiveness to acetylcholine (Ach) by enhancing epithelial acetylcholinesterase activation. However, the effect of estrogen on airway inflammation is still controversial. Other studies have shown that allergen-induced airway inflammation in ovariectomized rats decreased compared with intact females [33]. Moreover, de Oliveira et al. [34] indicate that estradiol increases pro-inflammatory cytokines IL-1β and TNF-α and decreases anti-inflammatory cytokines IL-10 in BAL cells in OVX-allergic rats. The present study demonstrated that estrogen had a protective effect against allergen-induced airway inflammation via mechanisms that included inflammatory cell recruitment; mucus production; increase in IgE level; multiplication of inflammatory cells in BALF; and production of pro-inflammatory cytokines, such as IL-6 and TNF-α, which were significantly reduced in mice treated with estrogen. The different protocols and animal models may cause different results.

NLRP3 inflammasome is widely considered to play an important role in allergic airway disease. An *in vivo* and *in vitro* study demonstrated that active caspase-1 staining was strongly expressed in OVA mice and NLRP3 and IL-1β protein expression was significantly increased in lipopolysaccharides (LPSs) priming normal human bronchial epithelial cells [19]. Furthermore, Besnard et al. [20] using mice with OVA-induced deficiencies in NLRP3, IL-1R1, IL-1β or IL-1α, indicated that NLRP3 activation was required in allergic airway inflammation, and that NLRP3 was associated with T_h_2 pro-inflammatory cytokines. Another study showed that NLRP3-specific inhibitor could reverse neutrophilic inflammation in allergic airway diesese [25]. Our data showed that NLRP3, ASC, and caspase-1 expression was increased after OVA challenge, further confirming that allergic airway inflammation was associated with activation of NLR signaling. Taken together, these studies indicate that pharmacological inhibition of NLRP3 might be beneficial for the treatment of inflammatory diseases, including asthma.

The next problem to be solved in our study was whether E2 regulated NLRP3 in allergic airway inflammation. E2 treatment has been reported to up-regulate NLRP3 activation in the brain. Xu et al. [26] indicate that E2 and ERβ agonists reverse NLRP3 activation caused by estrogen deficiency. Similarly, E2 has been reported to noticeably inhibit NLRP3 activation and pro-inflammatory cytokine production in the brain after global cerebral ischemia [27]. Moreover, E2 appears to attenuate the activation of NLRP3 via ERβ mediation in fibroblast-like synoviocytes (FLSs) [35]. Another study by Heitzer et al. [36] showed that E2 reduced NLRP3 protein, activate caspase-1 and mature IL-1β in mice with amyotrophic lateral sclerosis. However, the interaction between estrogen and NLRP3 in allergen-induced airway inflammation is still unknown. Our current study demonstrated that E2 treatment markedly inhibited the mRNA and protein expression of NLRP3, ASC, and cleaved caspase-1 in lung tissue. This implied that E2 might inhibit inflammation by decreasing NLRP3 transcription and activation. This is not consistent with a previous report that estrogen up-regulates NLRP3 via ERβ mediation in hepatocellular carcinoma cells [37]. Therefore, we considered that the regulatory effect of estrogen on NLRP3 might differ according to disease.

As mentioned above, the pro-inflammatory cytokines IL-1β and IL-18 are activated by cl-caspase-1 and then participate in airway inflammatory disease. IL-1β is capable of enhancing the production of immunomodulatory mediators, which are required for extracellular matrix (ECM) degradation and airway remodeling in asthma [38]. Meanwhile, IL-1β signaling is considered to play an essential role in polarization of IL-17-producing Th17 cells [39]. A study of neutrophilic asthma patients demonstrated that IL-1β production was significantly elevated, as was expression of NLRP3 and caspase-1 [40]. Likewise, our findings showed that IL-1β, but not IL-18, was increased in OVA-challenged mice. It seemed that E2 inhibited transcription of and reduced production of IL-1β. Meanwhile, a lot of studies suggested that IL-18 was associated with asthma severity. IL-18 was increased in asthmatic patients compared with that in healthy subjects [41,42]. However, there were still controversial researches showing that sputum supernatants [43] and serum [44] IL-18 were found at low levels in patients with severe refractory asthma (SRA). In our study, there was no difference in IL-18 expression, the reason may relate to the protocol of allergic airway inflammation model.

Classically, estrogen is activated by combining two nuclear estrogen receptors, ERα and ERβ, in target cells. ERα and ERβ have different biological functions in the lung [45,46]. Our study showed that ERβ protein expression was significantly reduced in OVA-challenged mice. This was consistent with the report that *ERβ* mRNA is decreased by OVA challenge, and that reduction in ERβ expression may play an important role in OVA-challenged inflammation [47]. Some studies using ERβ agonists and antagonists further demonstrate that estrogen modulation of NLRP3 activation is ERβ-dependent [26,35]. While our study did not detect which ER mediates E2’s effect on NLRP3, considering the elevation of NLRP3 and the reduction in ERβ in OVA-induced mice, we supposed that ERβ may play a protective role against allergic airway inflammation. Indeed, there are few studies about ERα expression of lung in OVA mice. We further look for literature related to ER and AHR. A study in human ASM demonstrated that in human ASM, ERα expression (calculated relative to GAPDH expression) was greater than that of ERβ. The study also found that estrogen reduced Ca^2+^ influx in ASM using Ach via ERα, further suggested estrogen relaxed ASM [14]. The other study indicated that mast cells expressed mRNA for ERα, but not ERβ. In addition, the study demonstrated that estrogen-activated mast cells using IgE allergens via ERα and Ca^2+^ influx [48]. However, these two studies focussed on specific cells and allergic airway diseases. The composition of lung tissue is very complex, including various cells and substances. In fact, our study has not classified specific cells at all, we hope the further study would be performed.

In conclusion, the results of the present study suggested that both mRNA and protein levels of NLRP3 were strongly increased after OVA challenge, and that E2 markedly suppressed airway inflammation and NLRP3 activation. Therefore, NLRP3 activation regulated by E2 appears to be a potential therapeutic target for allergic airway disease. Further studies are required to explain the mechanism by which estrogen regulates the activation of NLRP3.

## Abbreviations

AHR: airway obstruction and hyperresponsiveness
ASC: apoptosis-associated speck-like protein contaning CARD
ASM: airway smooth muscle
BAL: bronchoalveolar lavage
BALF: BAL fluid
ERα: estrogen nuclear receptor α
ERβ: estrogen nuclear receptor β
E2: 17β-estradiol
IL: interleukin
i.p.: intraperitoneal
PAS: Periodic acid–Schiff
TNF-α: tumor necrosis factor-α
